# Short-term memory retrieval enhances brain functional connectivity

**DOI:** 10.3389/fnbeh.2025.1578415

**Published:** 2025-04-30

**Authors:** Fanglei Duan, Xiangyu Yan, Jing Wang, Zhenhua Wu, Yixin Zhang, QiCheng Shu, Fangfang Liu, Fan Xu, Qin Han

**Affiliations:** ^1^Department of Pathology, Sichuan Clinical Research Center for Cancer, Sichuan Cancer Hospital and Institute, Sichuan Cancer Center, Affiliated Cancer Hospital of University of Electronic Science and Technology of China, Chengdu, China; ^2^Department of Evidence-based Medicine and Social Medicine, School of Public Health, Chengdu Medical College, Sichuan, China; ^3^Department of Clinical Medicine, School of Clinic Medicine, Chengdu Medical College, Sichuan, China; ^4^Department of Art, Art College, Southwest Minzu University, Chengdu, China; ^5^Department of Experimental Centre, School of Public Health, Chengdu Medical College, Sichuan, China

**Keywords:** fNIRS (functional near infrared spectroscopy), young adults, working memory, functional connectivity, short-term memory, cognitive neuroscience

## Abstract

**Introduction:**

Short-term memory poses a significant challenge, involving complex processes of image perception, memory formation, and execution. However, the mechanisms underlying the formation, storage, and execution of short-term memory remain poorly understood.

**Methods:**

In this study, 41 healthy college students participated in a memory challenge test designed to investigate these processes. Functional near-infrared spectroscopy (fNIRS) was employed to measure dynamic changes in hemoglobin concentrations in specific cortical regions, while facial expressions and vital signs were recorded in real-time during the tests.

**Results:**

The results revealed heightened activity in the inferior prefrontal gyrus, visual association cortex, pre-motor cortex, and supplementary motor cortex. Functional connectivity between these regions was significantly enhanced during the tasks, and inter-group differences decreased over time. Participants with superior short-term memory exhibited lower levels of negative emotional expressions and higher heart rates compared to those with weaker memory performance. These findings suggest that cortical interconnectivity and adequate cerebral blood oxygenation play critical roles in enhancing short-term memory capacity. This has important implications for education, as it highlights strategies for cultivating attention, training memory skills, and improving memory integration abilities.

## Introduction

Short-term memory refers to the ability to briefly retain and process information, serving as an initial input channel for working memory. It plays a pivotal role in cognitive functions by acting as a temporary information repository that allows us to remember auditory, visual, olfactory, or conceptual inputs for short durations. This ability is essential for acquiring new knowledge, making decisions, communicating effectively, and facilitating higher-order thinking processes. Unlike long-term memory, short-term memory has a limited capacity. George A. Miller, an American psychologist, proposed the “7 ± 2 rule,” suggesting that short-term memory can typically hold seven units of information, with a range of 5–9 (Ricker et al., 2016). As a gateway to long-term memory, short-term memory serves as a bridge between transient sensory inputs and more permanent storage, functioning as a filter to determine which information is worth encoding into long-term memory (Wen and Ong, 2024). Brown (1958) proposed that short-term memory decays rapidly over time, becoming unreliable after reaching a certain threshold (Ricker et al., 2016). The duration of short-term memory is generally brief, ranging from a few seconds to 1 min. Although incoming information can be encoded and organized during this period, it is highly susceptible to interference. For instance, new information or external distractions can easily disrupt and overwrite existing content. Visual information is initially processed in the lateral geniculate nucleus of the thalamus and transmitted to the primary visual cortex (V1) in the occipital lobe. From there, it undergoes further analysis in associated cortical areas, including V2, V3, V4, and V5. The hippocampus, in conjunction with the medial temporal lobe, consolidates memory and filters information for long-term storage. The frontal lobe plays a regulatory role by directing attention, selecting relevant memory cues, and initiating retrieval processes. Additionally, the primary motor cortex, anterior motor cortex, and parietal cortex contribute to dictation processes, with the anterior motor cortex preparing muscle movements and the parietal cortex guiding these movements for accuracy (Liuzzi et al., 2010).

Cortical regions involved in memory processing include the hippocampus (Lisman et al., 2017), temporal lobe (Wu and Buckley, 2022), prefrontal cortex (Rolls, 2022), and the amygdala (Roesler et al., 2021). Lane Yoderumiersearch revealed that certain neurons are consistently activated at high frequencies during short-term memory storage. These neurons exhibit five key features associated with memory formation, retention, retrieval, termination, and error correction (Yoder, 2024).Short-term memory formation begins with external sensory input, such as visual, auditory, or olfactory information, which is encoded in specific brain regions. For example, visual inputs are initially processed in the occipital lobe, while auditory inputs are encoded in the temporal lobe. After encoding, memories are organized and temporarily stored in the hippocampus, with the dorsal frontal cortex managing these processes and the anterior cingulate cortex helping to sustain focus. Retrieval of short-term memory involves activation of the medial temporal lobe and the left midbrain prefrontal cortex, with neuronal activity mechanisms such as dendrite retention and reading (DHR) playing a role (Santos et al., 2012). The hippocampus, prefrontal cortex, and medial temporal lobe structures collectively support short-term memory through intricate interactions (Kesner, 2007).

Moreover, emotional states can influence short-term memory performance. Negative emotions, for instance, consume cognitive resources and diminish focus, impairing the encoding and retention of information (Maximilien Labaronne et al., 2025; García-Pacios et al., 2015). In contrast, positive emotional states enhance short-term memory by increasing attention and neuronal excitability. Studies have shown that individuals with strong short-term memory exhibit elevated heart rates, suggesting improved cerebral blood flow and oxygenation, which facilitate neuronal activity and temporary information storage. However, external factors such as visual interference from “memory mask” grating or hypoxia can disrupt short-term memory. The hippocampus, known for its sensitivity to oxygen deprivation, is particularly critical for spatial and episodic memory (Kesner and Hopkins, 2001). Blood oxygen levels in the prefrontal and parietal cortex have also been observed to increase during memory tasks, which can be measured using functional imaging tools such as fMRI. While fMRI is highly accurate, its use is limited in daily life due to practical constraints. In contrast, portable fNIRS devices offer a more accessible method for directly detecting cortical hemodynamic activity and analyzing hemoglobin concentration changes in brain tissues.

Short-term memory is essential for performing complex tasks, as it is actively utilized during task execution (Panico et al., 2021). Despite its importance, few studies have examined differences in blood oxygen consumption across brain regions during short-term memory tasks, or the activation patterns and functional connectivity between these regions. To address this gap, we recruited 41 college students to participate in a memory challenge, where they memorized 18-digit numbers and performed four consecutive memory tests. Participants were divided into groups based on the number of digits memorized in each trial. Using fNIRS, the blood oxygen consumption was analyzed in various brain regions and assessed differences in functional connectivity strength across groups during short-term memory formation and decay. Additionally, real-time facial expression analysis tools and physiological measurements were used to track participants’ emotional and physiological states. Based on the above evidences, we hypothesized that the enhanced functional connectivity between parietal-temporal networks may exhibit positive covariation with regional cerebral blood oxygenation (CBrO2) levels, and their synergistic interaction may involve in mediate the enhancement of short-term memory capacity.

## Materials and methods

### Ethics statement

This study was approved by the Ethics Committee of Chengdu Medical College (Approval Number: 2023 No. 113). All procedures were non-invasive and non-contact, with professional staff supervising the entire process to ensure smooth operation and participant safety.

### Participants

The study recruited 41 healthy college students, including 11 males and 30 females. Each participant completed four memory tests, during which they were required to memorize an 18-digit sequence. A successful recall of 12 digits was used as the threshold to separate participants into two groups: Group A (recall ≤ 12 digits) and Group B (recall > 12 digits).

### Inclusion and exclusion criteria

Participants aged 18–22 years were included if they met the following criteria: good physical and mental health status, absence of serious neurological or systemic diseases affecting memory function, normal intelligence, fluent language proficiency, no history of substance or alcohol abuse, and willingness to provide written informed consent. Exclusion criteria comprised conditions potentially influencing memory performance, including acute medical events, anxiety, depression, neurological or psychiatric disorders, metabolic diseases, poisoning, drug abuse, active infections, or other systemic illnesses. Additionally, individuals with language barriers, cognitive impairments hindering questionnaire completion, or inability to accurately comprehend and respond to study requirements were excluded.

### Test procedure

To ensure consistency across participants, all experimental sessions were conducted in a temperature-controlled (22 ± 1°C), sound-attenuated laboratory with uniform lighting (500 lux). Participants were tested between 9:00 and 11:00 a.m. after abstaining from caffeine and alcohol for at least 12 h. Each Participant wore optical caps and were instructed to sit upright in front of a camera equipped with facial recognition functionality to ensure their faces were fully visible. Each test involved memorizing an 18-digit sequence within 1 min. After a 30-s pause, participants wrote down the digits on paper, followed by a 30-s rest period with their eyes closed. This process was repeated three additional times, with each round featuring a different 18-digit sequence.

Participants were divided into two groups based on their recall ability (≤ 12 digits for Group A and > 12 digits for Group B). In the experiment, the threshold for grouping was set at “remembering 12 numbers,” which is based on the core mechanism of the working memory capacity theory. According to Miller’s chunking theory (1956), the capacity of working memory was expressed as 7 ± 2 meaningful chunks rather than the raw number of digits. When the number of digits reaches 12 (if each chunk contains 2–3 digits, corresponding to 4–6 chunks), it is precisely at the critical interval of the “effective chunk capacity.” Those who can remember 12 or more digits indicate that they have exceeded the basic capacity limit through chunking strategies (such as encoding as dates or regular sequences); those who can remember less than 12 digits may rely on mechanical memory, reflecting insufficient chunking ability or lower efficiency in resource allocation. The resource allocation theory (Baddeley, 2000) further supports this threshold: cognitive resource consumption increases with the load of digits, and 12 serves as the upper limit of memory span after strategy optimization in behavioral research (Ricker et al., 2010; Ericsson, 2004), which not only conforms to the derivation of “4 core chunks × 3 digits/chunk” in Cowan’s revised theory but also aligns with the sudden increase in error rate in the pre-experiment, marking the saturation critical value of individual resource allocation (Norris and Kalm, 2021; Norris et al., 2020).

Four different sets of numbers, designed to be sufficiently challenging, were used to increase the experiment’s validity and effectiveness. The experimental timing was carefully structured to maintain a reasonable balance between compactness and operability, while still generating sufficient data for statistical analysis. In the first experiment, group A (*n* = 13) and group B (*n* = 28), in the second experiment, group A (*n* = 24) and group B (*n* = 17), in the third experiment, group A (*n* = 22) and group B (*n* = 15), in the fourth experiment, group A (*n* = 19) and group B (*n* = 18).

**FIGURE 1:**
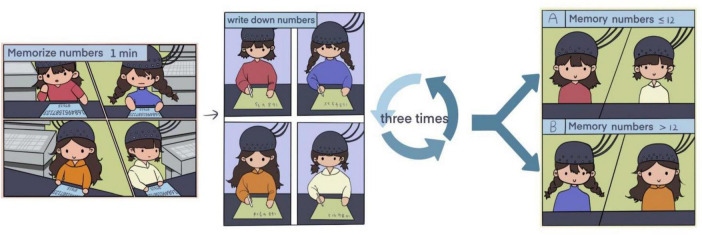
Experimental flow chart.

During the memory tasks, fNIRS and a video camera monitored fluctuations in HBO (oxyhemoglobin), HB (deoxyhemoglobin), and HBT (total hemoglobin) levels, as well as facial expressions and life signs across various regions of the cerebral cortex. The detailed workflow was illustrated in Figure 1.

### fNIRS data acquisition

**FIGURE 2:**
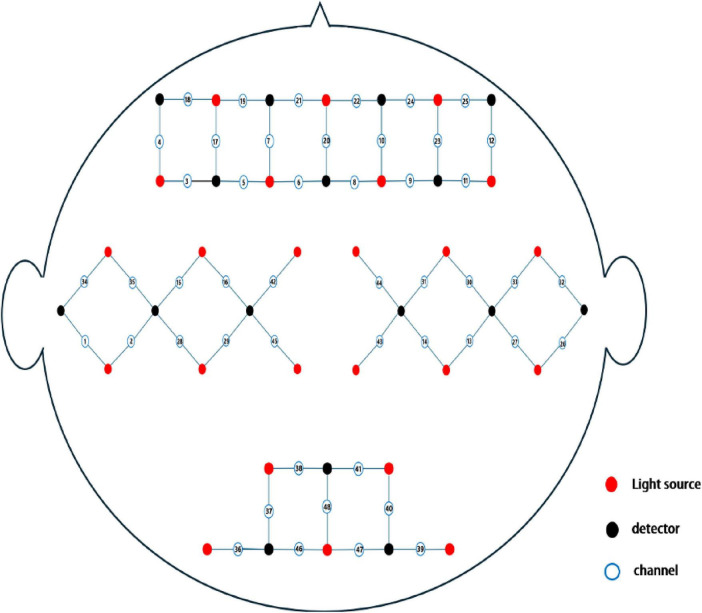
Schematic layout of fNIRS channels.

Cerebral cortex activity was recorded continuously using a multichannel fNIRS system (Danyang Huichuang Medical Equipment Co., Ltd.). The system included 24 transmitters and 16 receivers, forming 48 effective observation channels with an average transmitter-to-receiver distance of 3 cm. Each channel’s center was defined as the primary brain region detected, with brain localization performed using this reference point, details channels distribution (see Figure 2).

The NirSpark analysis system (Danyang Huichuang Medical Equipment Co., Ltd.) was employed to analyze brain functional connectivity, blood oxygenation trends, and differences in blood oxygen content between groups, detail channel distribution (see Supplementary Table 1).

### Facial expression and life sign measurement

A camera was used to capture participants’ full-face expressions, which were analyzed using the FaceReader Noldus 7.0 software. Life signs, including heart rate, SpOod oxytolic blood pressure (SBP), and diastolic blood pressure (DBP), were measured using the Mindray Life Surveillance System (VS-9).

### Statistical analysis

All data were stored and managed using Microsoft Office 365. Measurement data were expressed as mean ± standard deviation (SD). Life signs and facial expression data were compared using a two-tailed *t*-test, while fNIRS data between the two groups were analyzed using a one-way ANOVA. Statistical analysis and graphical visualizations were performed using Stata 18 MP software. A *P*-value of < 0.05 was considered statistically significant.

## Results

### Demographic

A total of 41 participants were enrolled in the study, comprising 30 females and 11 males, with an average age of 19.43 years. Participant demographics, including age and sex, were analyzed for each test group based on their recall performance. No statistically significant differences in age or sex were observed between Group A (recall ≤ 12 digits) and Group B (recall > 12 digits) across any of the four tests (see Table 1).

**TABLE 1:** Demographic background of participants.

Test	Subgroup	Group A (memory number ≤ 12)	Group B (memory number > 12)	*t*/*x*^2^	*p*
1	Gender [*n* (male%)]	6 (46.2%)	6 (20.4%)	2.622	0.105 > 0.05
	Age (years)	19.46 ± 1.19	19.42 ± 1.23	0.08	0.936 > 0.05
2	Gender [*n* (male%)]	7 (29.2%)	5 (29.4%)	0.001	0.986 > 0.05
	Age (years)	19.25 ± 1.03	19.70 ± 1.43	1.20	0.237 > 0.05
3	Gender [*n* (male%)]	8 (36.4%)	4 (21.1%)	1.154	0.283 > 0.05
	Age (years)	19.59 ± 1.29	19.26 ± 1.09	0.86	0.392 > 0.05
4	Gender [*n* (male%)]	7 (36.8%)	5 (22.7%)	0.981	0.322 > 0.05
	Age (years)	19.42 ± 1.16	19.45 ± 1.26	0.88	0.931 > 0.05

Based on the results of the four memory tests, a statistically significant difference was noted in the distribution of participants between Group A and Group B in the first test. However, no such differences were observed in the second, third, or fourth tests (see Table 2).

**TABLE 2:** Memory test results comparison.

Test	Subgroup	Group A (memory number ≤ 12)	Group B (memory number > 12)	*x* ^2^	*p*
1	Toll	13	28	5.90	<0.05
2	Toll	24	17	1.57	>0.05
3	Toll	22	19	0.38	>0.05
4	Toll	19	22	0.38	>0.05

### Time-dependent effects on blood oxygen concentration

**FIGURE 3:**
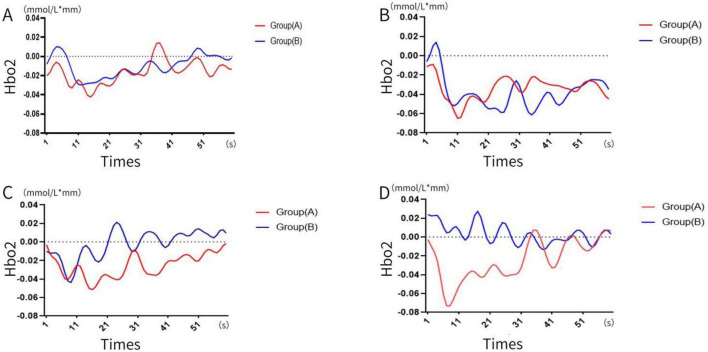
Change in blood oxygen concentration. **(A)** First time, **(B)** second time, **(C)** third time, **(D)** the four times. (A: memory numbers ≤ 12, B: memory numbers > 12).

**FIGURE 4:**
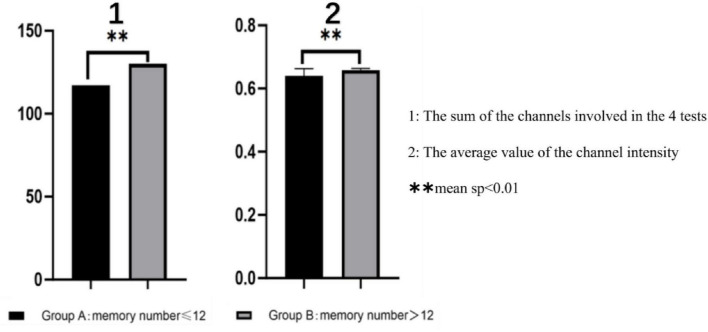
Functional connectivity difference, **sp < 0.01.

The dynamic changes in blood oxygen concentration during the four memory experiments were recorded and visualized (see Figures 3, 4). Results revealed that blood oxygen concentration in Group B (memory number > 12) was significantly higher than that in Group A (memory number ≤ 12) across all four tests.

### Visualization of functional connectivity during memory tasks

Functional connectivity strength was analyzed and visualized during the memory tasks for both groups. Group B (recall > 12 digits) consistently demonstrated significantly stronger functional connectivity in bilateral brain regions during all four experiments, whereas Group A (recall ≤ 12 digits) exhibited weaker connectivity. Across the four experiments, the total number of channels involved in functional connectivity was lower for Group A (117 channels) compared to Group B (130 channels). Furthermore, the average channel intensity was also lower in Group A (mean = 0.64) compared to Group B (mean = 0.66).

A one-way ANOVA was conducted to assess differences in brain functional connectivity strength between the two groups. Results showed statistically significant variations in channel connectivity across the four experiments: 18th channels exhibited significant differences in the first experiment; 20th channels exhibited significant differences in second experiment, 6th channels exhibited significant differences in the third experiment, while 10th channels exhibited significant difference in the fourth experiment.

**FIGURE 5:**
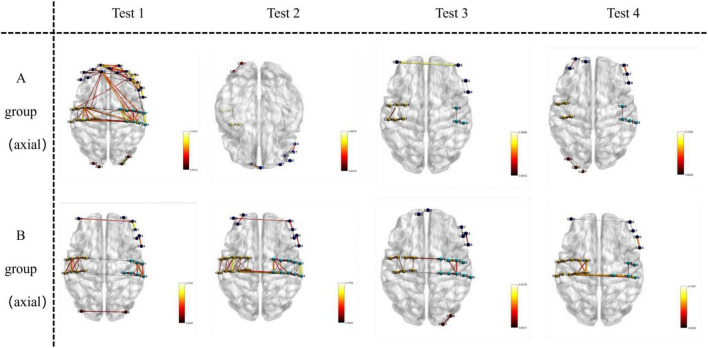
Visualizes functional connections in the axial plane (horizontal slice) of the brain. For Group A and Group B, images in Test 1–4 are oriented as axial views, where the horizontal orientation of the brain sections represents the axial—plane location.

**FIGURE 6:**
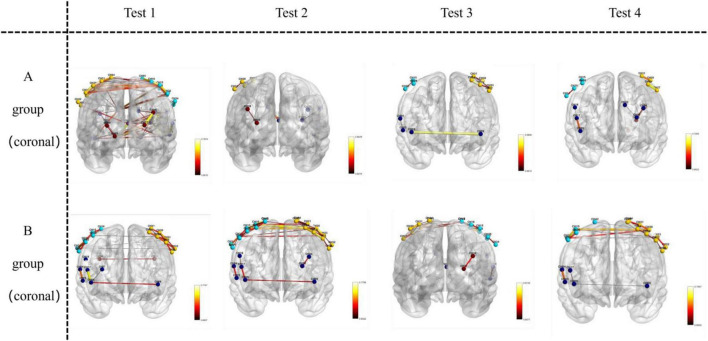
Visualizes functional connections in the coronal plane (front-to-back vertical slice) of the brain. For Group A and Group B, images in Test 1–4 are oriented as coronal views, where the front-to-back vertical orientation of the brain sections represents the coronal—plane location.

**FIGURE 7:**
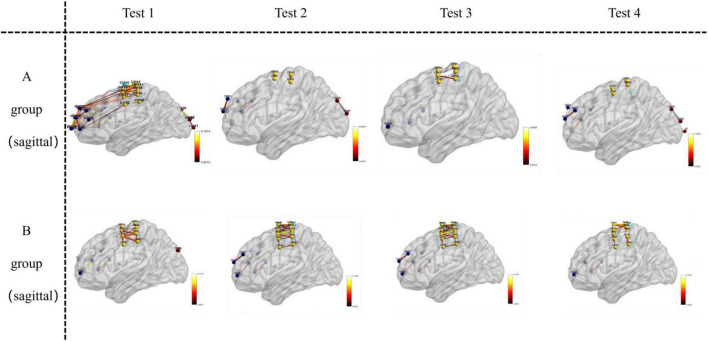
Visualizes functional connections in the sagittal plane (left-to-right vertical slice) of the brain. For Group A and Group B, images in Test 1–4 are oriented as sagittal views, where the left-to-right vertical orientation of the brain sections represents the sagittal—plane location.

Key brain regions involved in functional connectivity included the primary somatosensory cortex, primary motor cortex, pre-motor and supplementary motor cortex, dorsolateral prefrontal cortex, primary visual cortex (V1), and visual association cortex (V2–V5) (see Figures 5–7).

**FIGURE 8:**
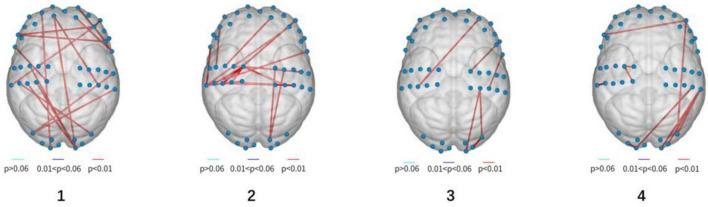
Visualization of functionally differentiated connectivity (from left to right, stands for experiment 1–4).

As the experiment progressed, the number of significant differences in functional connectivity between Group A and Group B gradually decreased (see Figure 8). For detailed values of ROI (region of interest) changes (refer to Table 3).

**TABLE 3:** Leverage functional connectivity values at the ROI level when remembering numbers.

ROI	Group A (memory number ≤ 12)	Group B (memory number > 12)
Pre-Motorex left∼ pre-motor right	0.30349	0.45538
Pre-Motorex left∼ PSC left	0.38303	0.55043
Pre-Motorex left∼ PSC right	0.32206	0.48664
Pre-motor right∼ PSC right	0.36150	0.56233
IPG left∼ PSC right	-0.03798	0.19817
Pre-Motorex left∼ pars triangularis Broca’s area left	0.17907	–0.4324
Pre-Motorex left∼ IPG right	–0.09547	0.116063
Pars triangularis Broca’s area right∼ DLPFC	0.25228	0.059618
Visual association cortex (V)’∼V	0.42016	0.22005
Visual association cortex (V)’∼ PSC left	0.33774	0.14826
Visual association cortex (V)’∼ PSC right	0.27756	0.11861

ROI, region of interest; DLPFC, dorsolateral prefrontal cortex; PSC, primary somatosensory cortex; PMC, Pre-motor and supplementary motor cortex; IPG, inferior prefrontal gyrus.

### Life signs

**FIGURE 9:**
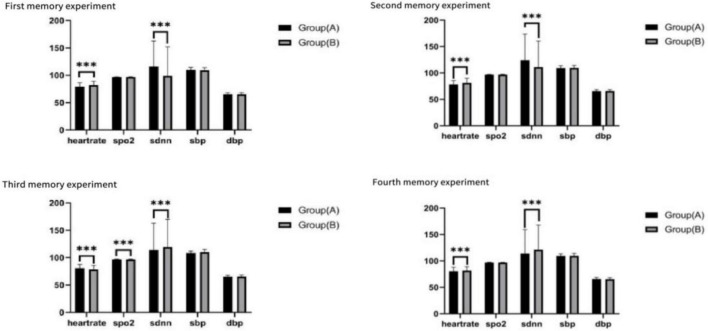
Life signs for the four tests, ***p < 0.01.

Significant differences were observed in heart rate and SDNN (standard deviation of normal-to-normal intervals) between the two groups across the four experiments. However, no significant differences were found for other physiological variables, including SpO2, SBP, and DBP (see Figure 9).

### Face expression

**FIGURE 10:**
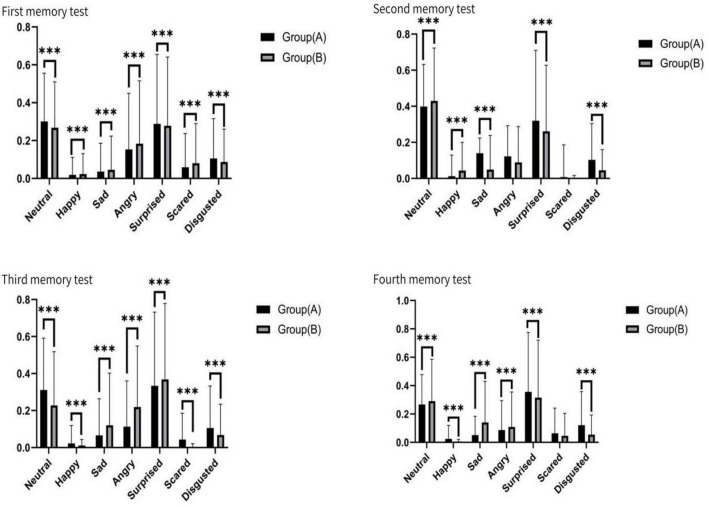
Face signs for the four tests, ***p < 0.01.

Across the four memory tasks, there was A statistically significant difference in expression between group A and group B. Neutral, angry and surprised expressions performed significantly better in the memory task than happy, sad, scared and disgusted. Neutral mood fluctuated slightly between the two groups. Angry emotion showed the quantitative effect of memory advantage, and group B performed better than group A. Surprised mood was stable and showed little difference between groups. Happy showed the weakest emotion and reached the lowest value on multiple tasks. Sad was more prevalent in group B. The Scared mood decreased as the test went on. Disgusted feelings were more pronounced in group A (see Figure 10).

## Discussion

**FIGURE 11:**
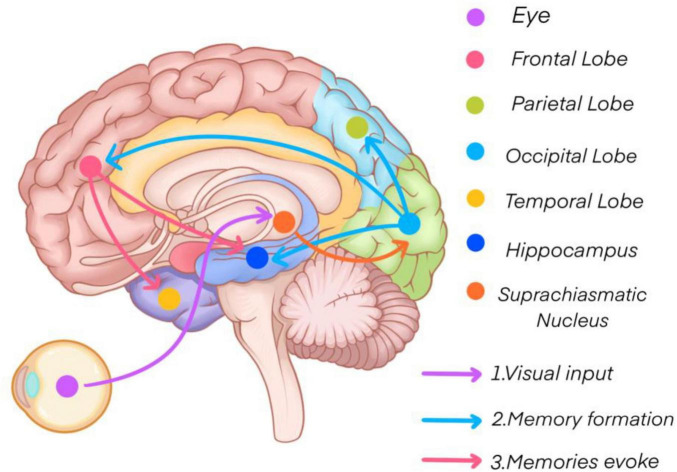
Short-term memory mechanisms.

This study utilized functional near-infrared spectroscopy (fNIRS) to investigate cerebral cortical activity and functional connectivity during short-term memory tasks, where participants were required to memorize four sets of 18 digits. The findings revealed that specific brain regions, including the lower frontal gyrus, visual association areas, premotor areas, and supplementary motor areas, exhibited elevated activity levels (see Figure 11). Additionally, participants with superior short-term memory performance demonstrated stronger functional connectivity between bilateral brain regions. Over the course of the experiment, functional connectivity within both groups increased, while the differences in connectivity between the two groups decreased. Vital sign data further indicated that participants with stronger short-term memory exhibited higher heart rates and lower levels of negative emotional expression.

The primary visual cortex, located in the occipital lobe, plays a crucial role in perceiving and temporarily storing external visual information, forming a visual transient memory. After attentional filtering, this information is encoded into short-term memory with the involvement of the prefrontal and parietal cortices (Phylactou et al., 2022). Subsequently, thorough processing in the hippocampus, medial temporal cortex, and frontal cortex transitions short-term memory into long-term memory by linking new knowledge to pre-existing information (Kesner, 2007; Qin et al., 2023).

Traditionally, short-term memory has been assessed using functional magnetic resonance imaging (fMRI). This technique focuses on activity within deep brain structures, such as the hippocampus and parahippocampal gyrus, which are significantly involved in short-term memory tasks (Voss et al., 2017). fMRI also provides insight into neural activity by measuring changes in glucose and oxygen metabolism (Xu et al., 2024). In comparison, fNIRS detects changes in oxygenated and deoxygenated hemoglobin concentrations in the superficial cortical regions of the brain (Pinti et al., 2020). While fMRI excels in examining deep brain structures, fNIRS offers higher temporal resolution, making it suitable for capturing the rapid cerebral hemodynamic changes associated with short-term memory (Shader et al., 2021). Both modalities can assess functional connectivity, but their mechanisms differ: fMRI measures changes in blood flow and oxygenation at specific brain regions, while fNIRS uses near-infrared light to detect fluctuations in hemoglobin concentration, reflecting cortical activity.

Interestingly, the results of this fNIRS study align with prior fMRI findings (Zhang et al., 2024), showing that individuals with stronger short-term memory demonstrate elevated oxygenated hemoglobin levels, indicative of heightened neural activity in relevant cortical regions. These elevated levels facilitate more efficient processing and integration of short-term memory tasks. Furthermore, individuals with robust short-term memory exhibit stronger functional connections among key brain regions, enabling faster and more effective information transfer.

Analysis of blood oxygen levels reveals distinct activation patterns between the initial two tests and the subsequent two tests. In the first and second tests, participants’ HBO levels exhibited a rapid increase at the onset of the task, reaching a peak within a short period, consistent with the typical cognitive activation pattern (Yeung and Han, 2023; You et al., 2023). Conversely, in the third and fourth tests, HBO levels demonstrated an initial decline, potentially attributable to fatigue induced by repeated task performance (Soltanlou et al., 2018). Across all four tests, HBO levels tended to stabilize or resume an upward trend after approximately 10 s, indicating that participants gradually adapted to the task demands. This observation aligns with the sustained resource investment model for working memory maintenance. Notably, blood oxygen levels were higher and more stable in the latter two tests compared to the first two, particularly evident in Group B. This suggests that repeated memory tasks may enhance short-term memory performance, with individuals possessing higher memory capacity demonstrating superior performance, corroborating previous research findings (Bui et al., 2013). Moreover, Group B (memory span > 12) consistently exhibited higher blood oxygen concentrations than Group A (memory span ≤ 12) across all four tests, underscoring the significant influence of cerebral blood oxygenation on short-term memory. Specifically, Group B showed early rapid responses and sustained high levels of HBO. Participants with strong short-term memory rapidly activated cerebral blood flow at the task onset, likely reflecting faster attentional focus and supporting efficient encoding of digit sequences (Wager and Smith, 2003). This finding is consistent with prior research demonstrating that individuals with higher working memory capacity exhibit a more pronounced increase in prefrontal HBO and a more rapid hemodynamic response during cognitive tasks, suggesting more efficient neurovascular coupling (Takeuchi et al., 2017). The sustained high HBO levels in Group B indicate a more consistent oxygen supply to the brain, which is consistent with the demands of the maintenance phase of working memory. Individuals with higher memory capacity may optimize neural resource allocation, thereby reducing metabolic fluctuations (Glimcher, 2013).

Functional connectivity patterns also differ between individuals with strong and weak short-term memory. Individuals with robust memory capabilities demonstrate close, efficient, and well-coordinated connections between relevant brain regions, especially between the parietal and frontal lobes, which are crucial for sustaining attention during memory tasks. Conversely, weaker functional connectivity in these regions can lead to memory failure or distraction. Dynamic changes in connectivity also occur during memory tasks, with strengthened connections between the prefrontal cortex, parietal lobe, and temporal lobe facilitating integration and processing of information.

The expression of emotion can regulate the difference of short-term memory. The significant advantage of angry and surprised expressions may be due to their preemptive occupation of short-term memory resources. Neuroimaging evidence suggests that angry expressions enhance the early encoding efficiency of visual working memory (increased α-band oscillation synchronization) within 200–300 ms by activating synergistic action between the amygdala and the dorsal anterior cingulate gyrus (Jackson et al., 2014). Surprise expressions may trigger attentional blink inhibition through the novel stimulus detection network (anterior insular-parietal joint area) and prolong the residence time of information in short-term storage (Vuilleumier et al., 2004). Intergroup fluctuations in neutral expressions may reflect the additional consumption of working memory by their ambiguous emotional representations. fNIRS studies have shown that the blood oxygen signal in dorsolateral prefrontal cortex (DLPFC) is significantly enhanced during neutral expression processing, suggesting the need to call on controlled attention resources for emotion classification. This “analytic cost” may crowd out the retelling capacity of short-term memory, resulting in the recall accuracy rate fluctuating with task context (Manelis et al., 2019). The persistent inefficiency of happy expressions correlates with the cognitive defocusing effect of positive emotions: fMRI data suggest that happy expressions activate the ventral striatum rather than the hippocampus, possibly suppressing detail binding through the dopaminergic reward pathway (Acevedo et al., 2014). The gradual attenuation of fear expression (β = -0.18) was consistent with the temporal sensitivity of short-term memory, where the strength of the amygdaloid-prefrontal functional connection decreased exponentially with repeated exposure, reflecting the rapid fading of threat signals without real consequences (Acevedo et al., 2014).

This study also controlled for several factors influencing short-term memory, such as age, sex, health status, and environmental conditions (Theofilidis et al., 2020). Statistical analyses confirmed that age and sex differences among participants were not significant. Other factors influencing short-term memory include the dominance of brain hemispheres: the left hemisphere is typically associated with verbal, semantic, and structured memory tasks, while the right hemisphere excels in spatial, visual, emotional, and context-based memory tasks. The two hemispheres work together to optimize short-term memory performance (Yin et al., 2022).

## Conclusion

Short-term memory performance is strongly influenced by functional connectivity and cerebral blood oxygenation. A stronger functional connection between the parietal and temporal lobes correlates with higher cerebral oxygen levels, which enhances memory capacity. Additionally, repeated memory tasks improve short-term memory performance while reducing inter-group differences in functional connectivity.

## Limitations

The sample size was relatively small, and the use of college students excluded individuals who may have discontinued their education, thereby reducing the generalizability of the findings.

## Data Availability

The raw data supporting the conclusions of this article will be made available by the authors, without undue reservation.
